# Fabrication and Mechanical Properties of High-Durability Polypropylene Composites via Reutilization of SiO_2_ In-Situ-Synthesized Waste Printed Circuit Board Powder

**DOI:** 10.3390/polym14051045

**Published:** 2022-03-05

**Authors:** Shenghui Tian, Baixue Li, Hui He, Xinlu Liu, Xin Wen, Zuolu Zhang

**Affiliations:** 1 Provincial and Ministerial Co-Constructive of Collaborative Innovation Center for MSW Comprehensive Utilization, School of Metallurgy and Materials Engineering, Chongqing University of Science and Technology, Chongqing 401331, China; tianshenghui@live.cn (S.T.); 2018005@cqust.edu.cn (X.L.); xinwen@cqust.edu.cn (X.W.); 2 Key Lab of Guangdong Province for High Property and Functional Macromolecular Materials, School of Materials Science and Engineering, South China University of Technology, Guangzhou 510640, China; libaixuedream@163.com; 3 Quality Inspection and Measurement Department, Tangshan Wenfeng Special Steel Co., Ltd., Tangshan 063299, China; zhzlu2000@163.com

**Keywords:** waste printed circuit board powder, polypropylene, hybrid, OIT, surface modification

## Abstract

This paper focuses on the characterization of the physico-chemical properties, surface modification, residual copper content and in situ hybrid inorganic particle modification of polypropylene (PP) composites reinforced by waste printed circuit board powder (WPCBP). A series of WPCBP/SiO_2_ hybrids (TSW) were prepared by a sol–gel method at different pH values. Characterization results revealed the in situ generation of SiO_2_ on the surface of WPCBP, and showed that with an increase in pH value, the size of SiO_2_ particles increased gradually and the copper content decreased in the TSW powder. The mechanical properties, oxidation induction time (OIT) and thermal properties of PP composites were improved by reinforcement with TSW, which might be ascribed to the formation of serrated interfaces. This work not only develops a powerful method to enhance the properties of PP/WPCBP composites, but also provides an environmentally sustainable approach to the high-added-value reutilization of WPCBP.

## 1. Introduction

According to United Nations estimations, the global output rate of e-waste has been growing by 10% every year and the total production of e-waste has reached more than 50 million tons annually [1,2]. An effective method to resolve the increasingly serious environmental pollution problems is to realize the recycling and reuse of these e-wastes. An emerging concern about the growing volume of end-of-life electronics has come into focus in recent years [3,4]. The metal in waste printed circuit boards (WPCBs) is easy to recycle and reuse, providing high economic benefits which have been effectively realized in industry [5,6], whereas the non-metallic portions of WPCBs are difficult to effectively reutilize owing to their complex composition, and are ultimately consigned to landfill or combustion [7]. The landfilling of waste residue not only occupies land resources, but also erodes soil and pollutes groundwater. In addition, the incineration of this waste produces toxic gases which are harmful to human health [1,8,9]. Therefore, it is particularly important to achieve the high-value recycling of WPCBP. The intrinsic complexity of WPCBP components makes their recovery very difficult, and their economic value is low [10,11]. Even so, the short glass fibers contained in WPCBP could be used as filler to improve the properties of a polymer matrix, providing a new alternative for recycling and reusing WPCBP while reducing production costs [12,13]. However, the chemical structures of WPCBP and WPCBP-reinforced polymer composites exhibit poor interfacial properties, narrowing the scope of their application in certain fields.

Therefore, it is becoming increasingly necessary to address the intractable problems of WPCBP-reinforced polymer composites to enable their rapid development and urgent application. Recently, hybrid filler fabricated with two geometrically dissimilar fillers was found to have significant synergistic effects in reinforcing a polymer matrix in comparison to the use of a single filler. Many efforts have been devoted to combination or hybridization with fillers, which is thought to represent a promising method of modifying composites and has sparked great interest among researchers and engineers [14,15]. Hybrid fillers incorporate two or more fillers through the interactions of chemical bonding, electrostatic adsorption and hydrogen bonding by self-assembly, wet chemistry, templating, or in-situ growth. Sol–gel synthesis is an effective method to prepare functional hybrid composites [16]. Researchers prepared cellulose/SiO_2_ hybrid composites using the sol–gel method by adopting TEOS as raw material. In addition, inorganic particles were deposited on the surface of the organic phase by hydrogen bonding to improve the thermal stability of microfibers [17]. Deterioration of the comprehensive properties of PP composites is attributed to the poor interface interaction between WPCBP and the PP matrix. The surface pre-treatment of WPCBP helps to improve the interfacial bonding between WPCBP and the PP matrix.

To further enhance the interfacial interaction of the WPCBP/PP blend, this work aims to generate SiO_2_ hybridization in situ on the surface of WPCBP through a sol–gel method. Moreover, the effects of hybrid filler on the structure and properties of PP composites are also investigated systematically in this work.

## 2. Materials and Methods

### 2.1. Raw Materials

The polypropylene resin (type PPH-T03, isotactic index ≥ 96.0, MFR = 3.0 g/10 min) was supplied by Maoming Branch of Sinopec. The WPCBP used in this work was 80 mesh and purchased from Qingyuan Jintian Enterprise Co., Ltd. (Qingyuan, China). PP-g-MAH with a grafting rate of 1.0% was obtained from Kingfa Scientific and Technology Co., Ltd. (Guangzhou, China). Silane coupling agent KH550 (chemically pure) was supplied by Jianghan Oilfield Co., Ltd. (Qianjiang, China). Dibutyltin dilaurate (DBTDL) was purchased from Guangdong Wengjiang Chemical Reagent Co., Ltd. (Guandu, China). Tetraethyl orthosilicate (TEOS), sodium dodecyl benzene sulfonate (SDBS), absolute ethanol and ammonia were of analytical grade. They were purchased from Guangzhou Chemical Reagent Factory (Guangzhou, China) and used as received without further purification.

### 2.2. Sample Preparation

#### 2.2.1. Preparation of WPCBP with In Situ-Formed SiO_2_ Hybrid

**Figure 1:**
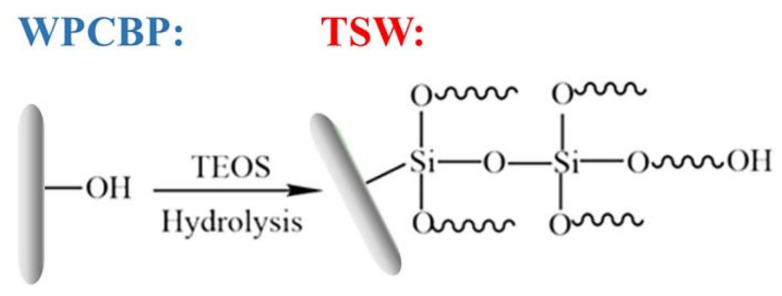
Schematic of the reaction between TEOS and hydroxy group in WPCBP.

The synthesis mechanism of WPCBP with in-situ-formed SiO_2_ hybrid (WPCBP/SiO_2_, abbreviated as TSW) is shown in Figure 1. First, WPCBP was dried in an oven for 2 h at 105 °C, then was screened through 80 mesh for standby. Certain amounts of TEOS were dispersed in anhydrous ethanol for 10 min, then a certain amount of deionized water was added and the mixture was mixed under ultrasonic treatment for 5 min. Then, the resulting solution was poured into a flask, and the pretreated WPCBP and ammonia were added. The mixture was stirred at a speed of 300 r/min for 3 h. The TSW hybrids were collected by filtration washing and freeze-drying. The pH values of the solution were kept in the range of 9–13, which maintained the sol solution in a more stable condition. TSWx stands for the WPCBP with the in-situ-formed SiO_2_ hybrid, where x represents the pH value during synthesis.

#### 2.2.2. Preparation of PP Composites

PP, WPCBP hybrid filler and PP-g-MAH (20 wt% of filler dosage) were mixed in a certain proportion, the melt compounding was carried out at 160 (+3) °C for 7 min using an open mill (XKR-160A, Zhanjiang machinery plant, Guangdong, China). Then, the samples were compression-molded for 3 min using a flat vulcanizer (XLB-D, Zhejiang Hongtu Machinery Factory, Zhejiang, China) with the temperature of the upper and lower plates at 180 °C, followed by the cold-press process for 7 min. After resting for 24 h, samples were cut off for standard test strips according to universal evaluation criterion.

### 2.3. Testing and Analysis

#### 2.3.1. TGA Analysis

TGA analysis was performed on a TA q5000 at a heating rate of 10 °C/min. The temperature ranged from 30 °C to 900 °C under dry nitrogen with a flow rate of 50 mL/min.

#### 2.3.2. Mechanical Properties

Tensile testing was conducted on a Zwick/Roell Z010 universal mechanical testing machine with a tensile rate of 20 mm/min according to the GB/T1040.1-2006 standard.

Flexural testing was performed on a Zwick/Roell Z010 universal mechanical testing machine with a span of 64 mm and an indenter rate of 20 mm/min according to the GB/T 9341-2008 standard.

V-notch impact testing was estimated using a 5113 digital impact testing machine according to ASTM D256 (Zwick company, Ulm, Germany).

#### 2.3.3. X-ray Fluorescence (XRF)

X-ray fluorescence analysis was collected on an Axios pw4400/40 XRF instrument with a high transmittance, SST ultra-sharp, long-life, ceramic end window (75 μm) rhodium target X-ray tube. The working conditions of power, maximum excitation voltage and maximum current were 4.0 kW, 60 kV and 120 mA, respectively.

#### 2.3.4. Scanning Electron Microscopy (SEM)

Morphologies and structures of samples were carried out with a Quanta 200 environmental scanning electron microscopy (SEM) machine (FEI, Eindhoven, The Netherlands) with the voltage of 10~15 kV.

#### 2.3.5. Oxidation Induction Time (OIT)

Testing of the oxidation induction time of composites was performed according to ISO 11357-6:2008 standard using a differential scanning calorimeter (DSC) (TA Instruments, New Castle, DE, USA) and thermo-gravimetric analysis measurement (TA Q20). A sample of about 5 mg was held at a temperature of 30 °C for 5 min under dry nitrogen, then the temperature was increased at a rate of 20 °C/min. Meanwhile, a sample was treated at constant temperature under oxygen atmosphere to record the oxidation induction time. The OIT end point was determined by the intersection of the tangent of the exothermic turning point and the constant-temperature baseline.

#### 2.3.6. Vicat Softening Point Analysis

The Vicat softening temperature of the PP composites was tested by a Vicat temperature tester HDT 3VICAT (CEAST, Turin, Italy) according to ISO 75-2:2003 standard, for characterization of the heat resistance of the composites. The experimental test conditions were as follows: 49.05 N loaded, the heating rate was 120 °C/h, and the liquid heat transfer medium was silicone oil.

## 3. Results and Discussion

### 3.1. Characterization of TSW

Ammonia was used as an alkali shrinkage agent to synthesize SiO_2_ in situ on the surface of WPCBP by the TEOS method (Figure 1). A series of TSW powders were prepared with different pH values. The micro-morphology and thermal stability of hybrid fillers were studied by means of SEM and TGA.

#### 3.1.1. Morphology Analysis of TSW

**Figure 2:**
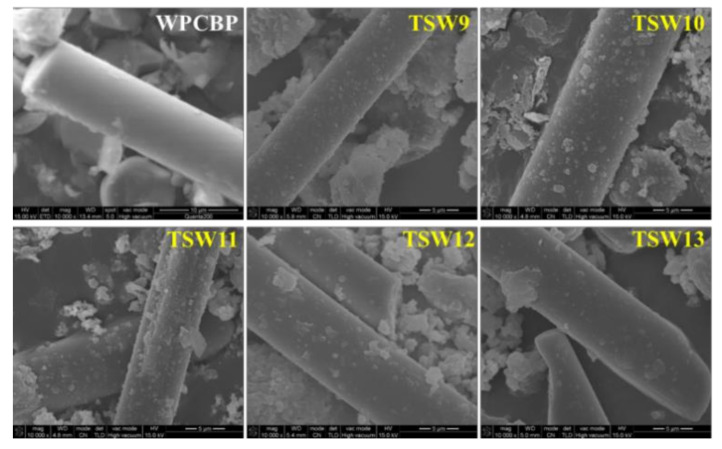
SEM photographs of different TSW hybrids (magnification: 10,000 times).

As illustrated in Figure 2, the surface of the primitive WPCBP was smooth without any obvious protrusions. After TEOS modification, the surface of hybrid filler became rough with the appearance of granular substances, indicating the in-situ generation of SiO_2_. In particular, the particle size of SiO_2_ on the surface of hybrid filler TSW increased gradually upon increasing the pH value, but the quantity of SiO_2_ was enhanced then suppressed. This phenomenon may have been due to the strong alkalinity which can intensify the degree of self-condensation of TEOS in the process of alkali condensation for the preparation of SiO_2_ [18,19]. As a result, the deposits of powder on the material surface were large and irregular, and the particle size also increased. In addition, the possibility of reaction between SiO_2_ and the hydroxyl group of WPCBP may be decreased with the intensification of self-condensation [20], resulting in the powder volume on the material surface first increasing and then decreasing.

#### 3.1.2. TGA Testing of TSW

**Figure 3:**
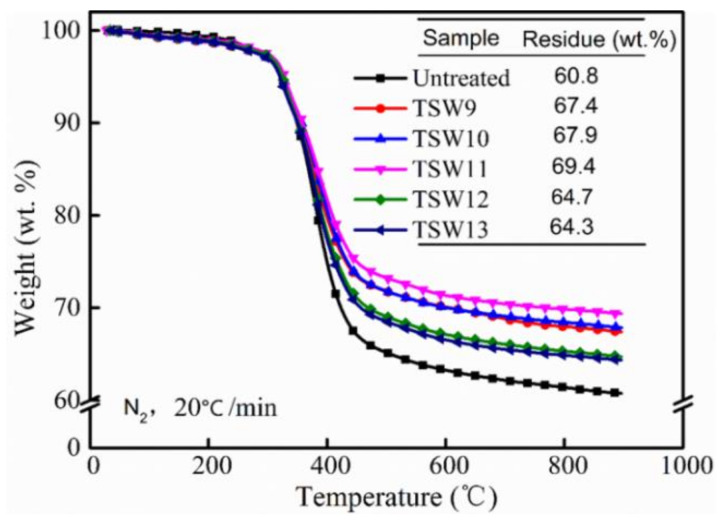
TGA curves and related char residue data for different TSW hybrids.

TGA tests were carried out to determine the thermal stability and char-forming capability of WPCBP and TSW samples. Figure 3 shows the TGA results for TSW produced with various pH values. The thermal stability and char residual weight of the hybrid filler were improved by the modification with TEOS. This improvement was due to the increase in the residual carbon rate of the material resulting from the increase of inorganic phase in the hybrid filler. Moreover, with the increase in pH value, the residual weight of the powder first increased and then decreased, which was consistent with the results in Figure 2, indicating that the hybridization of SiO_2_ first increased and then decreased. The residual char weight of the TSW11 system reached the highest level of 69.4 wt%, suggestive of the highest amount of SiO_2_ hybridization and indicating that a high loading TSW was generated in comparison to the results of previous studies [21,22].

#### 3.1.3. Morphological Analysis of Reaction Process

**Figure 4:**
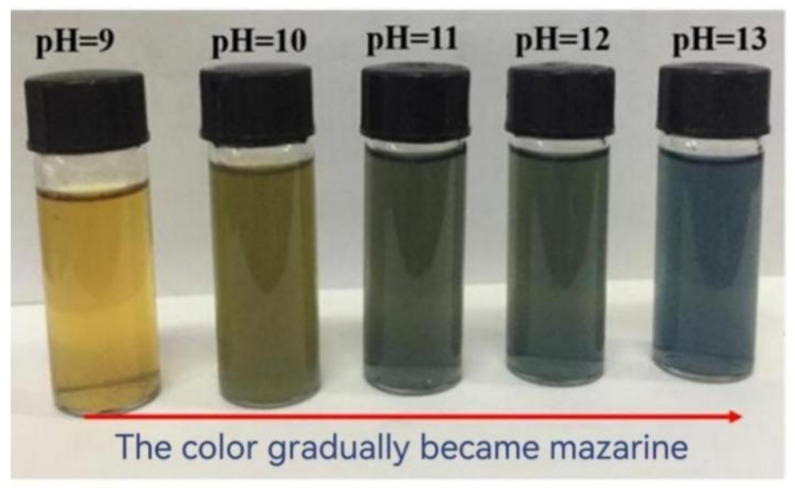
Digital photographs of different TSW filtrates.

Note that ammonia was used as the condensation agent for the preparation of TSW hybrids, which could promote the hydrolysis and condensation of TEOS [22]. The color change during the reaction was captured and the copper content in different TSW powders could be perceived simultaneously, as displayed in Figure 4. Upon increasing the pH value, the color of the TSW filtrate gradually became mazarine, manifesting the higher content of copper ammonia ion, and the calculation was further confirmed by the follow-up XRF characterization.

**Figure 5:**
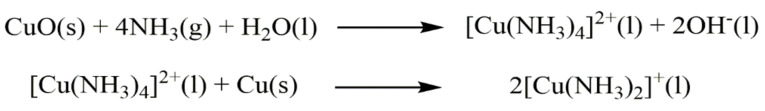
Schematic chemical color reactions of Cu.

In fact, the small quantities of residual metals (e.g., copper, iron, nickel) in WPCBP might cause side reactions with ammonia during the condensation process. As shown in Figure 5, the residual metal copper would form a blue complex with ammonia (the reaction being a color reaction), then the complex would continue to react with elemental copper to remove copper.

#### 3.1.4. XRF Analysis of TSW

WPCBP and TSW13 were treated in a muffle furnace at 700 °C for 4 h separately, and the residue contents of the samples were 62.1 and 65.4 wt%, respectively. The XRF measurement was able to quantitatively analyze the type and content of metal elements in their ashes, as listed in Table 1. The silicon content in the ash of WPCBP was more than that of TWS13, suggestive of the in situ formation of SiO_2_. Moreover, it can also be seen from Table 1 that the content of the variable-valence metal copper in the residue of the hybrid powder was reduced significantly. In addition, the reaction of the variable-valence metals in WPCBP with ammonia during the hybridization process will help to improve the weather resistance in PP composites doped with hybrid fillers [23].

### 3.2. Oxidation Induction Time of PP Composite

**Figure 6:**
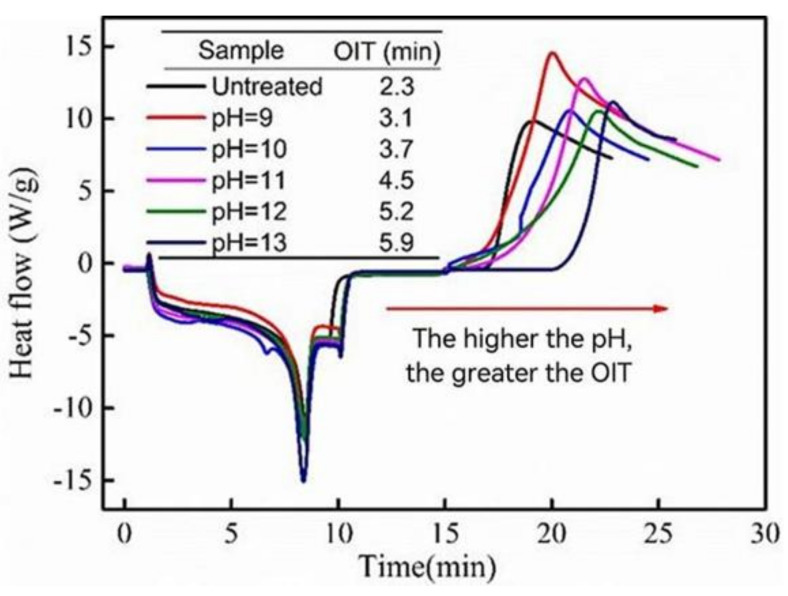
OIT curves of different TSW reinforced PP composites.

Figure 6 shows the effect of different TSWs on the OIT values of PP composites. With the increase of pH value, the OIT values of PP composites extended gradually and reached the maximum of 5.9 min at pH 13. These behaviors might be ascribed to the enhanced bonding ability between WPCBP and the PP matrix due to the in-situ hybridization of SiO_2_. On the other hand, with ammonia as promoter during the complexing reaction, the content of transition metals in the resultant TSW powder was low, which can also increase the OIT values of modified PP/TSW composites [24,25]. These findings are also consistent with the color change results in the hybrids filler solution and the XRF analysis of the hybrids.

### 3.3. Mechanical Properties of PP Composites

We studied the effects of TSW hybrids generated under various pH values on the mechanical properties of PP composites. It can be clearly seen from Table 2 that the mechanical properties of PP/TSW composites, including tensile strength, bending strength and bending modulus, were enhanced to various degrees compared to virgin PP and untreated PP/WPCBP composite. In the TSW13 system the tensile strength of 46.7 MPa and bending strength of 82.9 MPa were 38% and 26.5% higher, respectively, than those of the unmodified sample. This is probably because the uniform load of in-situ-synthetized SiO_2_ powder on the WPCBP surface might facilitate the formation of a serrated structure on the contact interface between WPCBP and PP. When the composite material is subjected to external force, these irregular surfaces could effectively prevent the growth of cracks and greatly enhance the mechanical properties of the composites.

The impact strengths of all hybrids system were higher than those of the unmodified sample, and the value for the TSW10 system was 3.67 kJ/m^2^, which was 28.8% higher than that of the unmodified system. In addition, the impact strength of PP/TSW composites first increased and then decreased with the increase in pH values during the hybrid reaction. These behaviors might be ascribable to the homogeneous distribution of SiO_2_ particles on the surface of WPCBP with low pH values, whereas relatively irregular dispersions were observed with high pH values owing to the large size of particles (Figure 2). The defects with large particle size were more likely to cause stress concentration, leading to the decrease in the impact performance of the composites.

### 3.4. Mechanism of Interface Analysis in PP Composite

**Figure 7:**
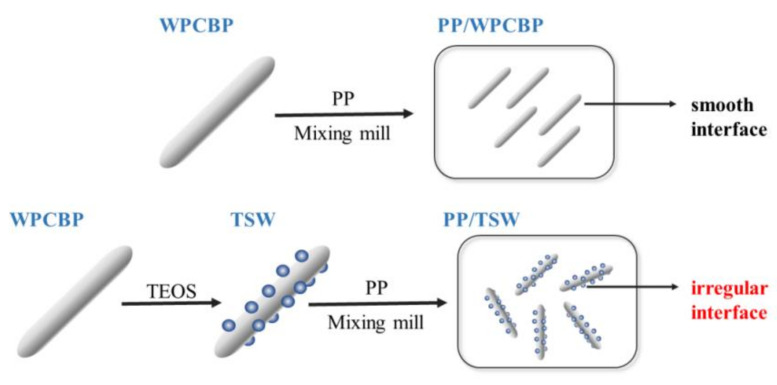
The proposed mechanism model of TSW reinforced PP composites.

The interfacial adhesion between the filler and the polymer matrix had a great effect on the mechanical, thermal and rheological properties of materials, and a superior interfacial adhesion between filler and matrix was conducive to the improvement of comprehensive material properties. On the basis of the above analysis, a reasonable mechanism could be proposed and is described in Figure 7. 

Figure 7 shows a feasible mechanism of PP composite modification by TSW. The specific surface area of the filler increased after in-situ hybrid modification, which increased the contact surface area between the hybrids and plastic matrix because of the formation of an uneven saw-tooth structure between filler and matrix. When the material was subjected to external force, the stress exerted on the PP matrix could be effectively transmitted to the hybrid portion. Furthermore, the interface could also efficiently transfer stress to reduce the probability of cracking along the glass fiber surface. Hence, the mechanical properties of composites were improved distinctly after hybrids modification.

### 3.5. Morphology of PP Composite

**Figure 8:**
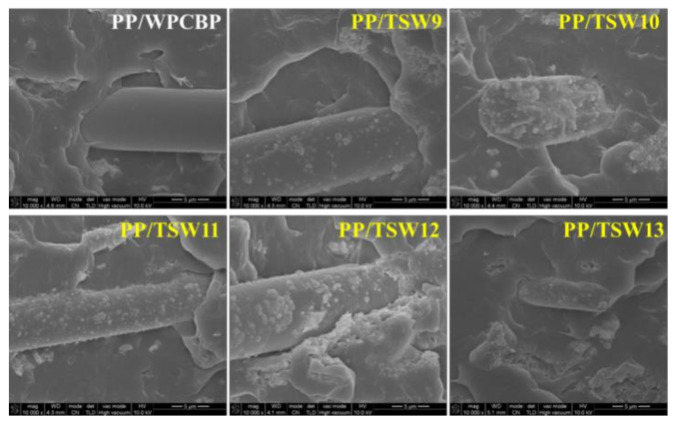
SEM photographs of TSW reinforced PP composites (magnification: 10,000 times).

SEM photographs of different TSW reinforced PP composites were shown in Figure 8. It could be clearly seen that the smooth surface of unmodified WPCBP made a flat contact surface with the PP matrix, in which the glass fiber in the powder was detached, indicative of the poor interface bonding ability between the WPCBP filler and the PP matrix. This complex structure was detrimental to the stress transfer when the composite was subjected to external force, which resulted in deterioration of the mechanical properties of the material. The surface of WPCBP with the in-situ-synthesized SiO_2_ system was rough, and there were many granular substances on the contact interface between filler and matrix, forming a serrated contact surface, and most of the hybrid filler was embedded in the PP matrix. The results were consistent with the mechanical property measurement (Table 2) and the proposed modification mechanism (Figure 7).

It can also be seen from Figure 8 that with an increase in pH values of the hybrid reaction system, the amount of SiO_2_ on the surface of the hybrid in the PP composite first increased and then decreased, which is consistent with the mechanical property results in Table 2. Obvious cracks can be observed on the contact surface between the hybrid and PP matrix when the pH value of the reaction system were higher than 11, which can be attributed to the excessive SiO_2_ particle size in the hybrid. This caused stress concentration in the PP matrix under the external force, and resulted in large cracks which had a great impact on the interface bonding of the hybrid and the PP matrix, causing a decline in the impact performance of the material.

### 3.6. Thermal Properties of PP Composites

#### 3.6.1. Vicat Softening Temperature

Table 3 indicates the effect of hybrid reactions with different pH values on the thermal stability of different PP composites. Vicat softening temperatures of all hybrid systems were higher than that of the unmodified system, and with the increase of pH values, the Vicat softening temperature of the PP/TSW composites first increased and then decreased. The TSW11 system had the largest increase among all the samples, with the Vicat softening temperature reaching 118.5 °C. These results were mainly due to the generation of silica, which increased the inorganic phase in the composite system, improving the interfacial bonding ability between filler and matrix. Thus, PP/TSW composites possessed good thermal resistance with fine transfer of heat pressure during heating.

#### 3.6.2. TGA

**Figure 9:**
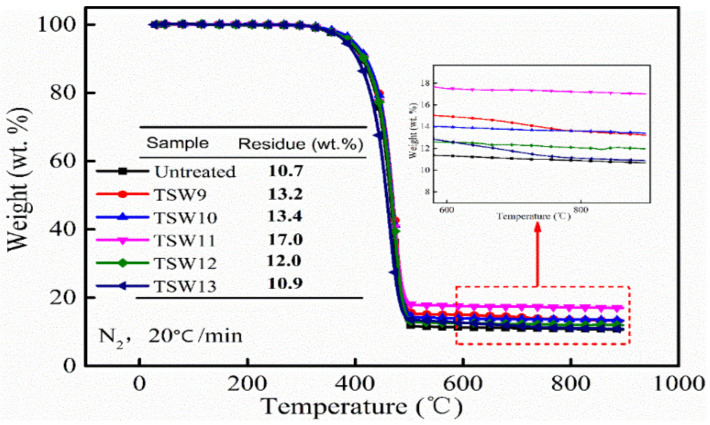
TGA curves and related char residue of different TSW reinforced PP composites.

Figure 9 shows the thermal stability of different PP/TSW composites. The residual weights of TSW-modified composites were higher than that of the original PP/WPCBP composite, and reached the highest PP/TSW11 value (17.0 wt%), which was attributed to the good thermal stability of in-situ-produced SiO_2_. Therefore, the char residue rate of the modified PP composites also increased. With the increase in pH value during the hybrid reaction, the residual weights of PP composites initially increased and then decreased, confirming that the amount of SiO_2_ first increased and then decreased. 

## 4. Conclusions

A series of WPCBP/SiO_2_ hybrids under different pH values were successfully fabricated using the sol–gel method. SiO_2_ was successfully synthesized in situ on the surface of WPCBP, and the size of SiO_2_ particles increased gradually with increasing pH. The copper content of TSW powder was significantly lower than that of archetypal WPCBP. The effect of TSW filler on the structure and properties of PP plastic was systematically investigated. The OIT of PP/TSW composite was high, with a maximum time of 5.9 min in PP/TSW13. Furthermore, increasing the hybrid reaction pH value was shown to enhance the tensile strength, flexural strength and flexural modulus of TSW composite material. Notably, the tensile strength and flexural strength of PP/TSW13 were respectively 38% and 26.5% higher than those of the unmodified system. In addition, the impact strength of the complex was 28.8% higher than that of its untreated counterpart owing to the serrated interface between TSW10 and the PP matrix. This strategy not only provides a novel method for improving the durability and mechanical properties of WPCBP-reinforced polymer composites, but greatly extends the comprehensive reutilization of WPCBP for a sustainable world.

## Figures and Tables

**Table 1:** XRF data of WPCBP and TSW13 after incineration.

Sample	Ingredient Content (wt%)							
	SiO_2_	CaO	Al_2_O_3_	MgO	CuO	Fe_2_O_3_	Cr_2_O_3_	Others
WPCBP	53.3	21.2	15.1	0.8	2.0	0.9	0.8	5.9
TSW13	61.2	16.7	12.9	0.6	0.7	0.8	0.8	6.3

**Table 2:** Effect of TSW hybrids on mechanical properties of PP composites.

Sample	Notched Impact Strength (kJ/m^2^)	Tensile Strength (MPa)	Flexural Strength (MPa)	Bending Modulus (GPa)
Virgin PP	4.51(0.27)	37.8(0.75)	57.3(1.65)	2.42 (0.13)
Untreated	2.85 (0.35)	33.4 (0.8)	64.9 (1.1)	2.73 (0.09)
TSW9	3.51 (0.32)	38.7 (1.2)	73.9 (1.7)	2.98 (0.13)
TSW10	3.67 (0.21)	42.7 (1.5)	77.9 (0.8)	3.07 (0.11)
TSW11	3.59 (0.37)	44.9 (0.7)	80.5 (1.4)	3.18 (0.07)
TSW12	3.21 (0.45)	46.1 (0.8)	82.1(1.5)	3.31 (0.05)
TSW13	2.88 (0.38)	46.7 (1.1)	82.9 (0.7)	3.37 (0.12)

**Table 3:** Vicat softening temperature of different TSW reinforced PP composites.

Sample	Untreated	TSW9	TSW10	TSW11	TSW12	TSW13
VST (°C)	113.1	114.6	118.1	118.5	115.3	115.9

## Data Availability

The data presented in this study are available on request from the first author S.T.
